# Effects of Supplementing Sea Buckthorn Leaves (*Hippophae rhamnoides* L.) and Chromium (III) in Broiler Diet on the Nutritional Quality and Lipid Oxidative Stability of Meat

**DOI:** 10.3390/antiox11112220

**Published:** 2022-11-10

**Authors:** Mihaela Saracila, Arabela Elena Untea, Tatiana Dumitra Panaite, Iulia Varzaru, Alexandra-Gabriela Oancea, Raluca Paula Turcu, Petru Alexandru Vlaicu

**Affiliations:** 1 Feed and Food Quality Department, National Research and Development Institute for Biology and Animal Nutrition, Calea Bucuresti, No. 1, 077015 Balotesti, Romania; 2 Nutrition Physiology Department, National Research and Development Institute for Biology and Animal Nutrition, Calea Bucuresti, No. 1, 077015 Balotesti, Romania

**Keywords:** chicken, chromium picolinate, nutrients, meat quality, oxidative stability, sea buckthorn leaves

## Abstract

Nowadays, the consumer trend towards healthier food choices is unquestionable. Meat products enriched with nutrients, such as polyunsaturated fatty acids and antioxidants, are gaining much more interest among consumers. However, products are susceptible to quality deterioration and a short shelf-life of meat through lipid oxidation due to the lack of antioxidants in the meat. In this regard, the efficacy of dietary sea buckthorn leaves (*Hippophaë rhamnoides* L.) together with Chromium on the nutritional quality of meat and lipid oxidative stability was investigated. An experiment (28 days long) was carried out on 90 Cobb 500 chickens assigned into three treatments: a control treatment based on corn and soybean meal, without Chromium (T0) and two treatments supplemented either with 0.00002% Chromium (T1) or with 0.00002% Chromium and 2% sea buckthorn leaves (T2). Dietary supplementation of SBL and Cr improved the PUFA/MUFA ratio, DHA concentration and decreased the n-6/n-3 ratio compared to the other treatments. Moreover, the breast and thigh meat belonging to T1 and T2 treatments showed a higher concentration of lutein and zeaxanthin, Fe and Zn and expressed a higher antioxidant capacity compared to those from T0. Furthermore, n-6 and n-3 PUFA deposited preferentially in the thigh meat rather than in the breast meat. The results from the study showed that dietary SBL and Cr significantly improved the fatty acid pattern and the oxidative stability of chicken breast meat, lowering the TBARS level after storage. In conclusion, SBL and Cr are promising dietary bioactive compounds with beneficial effects to obtain nutrient-enriched meat products.

## 1. Introduction

After improvements in the standard of living and in the context of the COVID-19 crisis, the concern/awareness of the population about the nutritional quality and health benefits of food has increased. Antioxidant compounds, such as vitamins, minerals, polyphenols, etc. are among the most-consumed nutrients in the form of supplements. As a result, there is a strong interest in developing healthier products, especially those that are easily accessible and frequently consumed (regularly). For example, in addition to being the main source of protein in a diet, meat can provide other bioactive compounds, such as vitamins, minerals, etc. In recent years, many efforts have been made to enrich meat products with nutrients required for a balanced diet. In the poultry meat industry, relevant nutritional findings play an important role in developing healthier meat products that provide the needed nutrients in the required amounts.

Compared to red meat, which is associated with a higher risk of metabolic diseases and cancer, chicken is considered a healthy and low-calorie meat for which there are no religious restrictions [1,2]. In this context, the consumption of chicken meat together with fish is recommended to have a balanced diet and reduce the risk of cardiovascular diseases and their risk factors, such as obesity and insulin resistance [3,4]. Despite the growing awareness of their health benefits, the intake of nutrients such as LC-PUFA remains below the recommended levels in many countries [5]. Considering these aspects, poultry seems to be a good matrix for food enrichment. Many studies have demonstrated the successful enrichment of meat with nutrients, such as polyunsaturated fatty acids, fat-soluble vitamins, minerals, etc. [6,7], when they added various plants/plant extracts or oils to the chickens’ feed. Dietary supplements from natural sources are appreciated by consumers who consider them safe and nutritious.

Sea buckthorn (*Hippophaë rhamnoides* L.) is a shrub known for its nutritious fruits or seeds that are consumed by people in various forms, from raw to juices, jams, oils, etc. Although sea buckthorn leaves (SBL) are not as well-known and used as the fruits, they are a source with a high content of active ingredients that can be included in the formulation of chicken feed to produce meat with added nutritional value. Sea buckthorn leaves are also rich in various valuable compounds, such as antioxidants, fiber, fatty acids, minerals, etc. [8,9,10], which have antioxidant and antimicrobial activities [9,11,12]. Such bioactive compounds can be used in animal feed to obtain meat products enriched with nutrients. However, the development and marketing of enriched meat products can be very challenging compared to conventional products, which have a high health image. Meat-enriched products represent a category of remarkably promising foods with beneficial properties, such as cholesterol-lowering, antioxidant, and anti-cancer properties that are considered very attractive by consumers [13]. The use of sea buckthorn leaves (waste/by-products of harvest) in chicken feed can be a practice to obtain value-added animal products and contribute to the circular economy [14]. In the light of the fact that food waste is a global problem [15], using all parts of vegetables and fruits is a favorable/adequate/sustainable solution. Although most studies have been conducted on fruits, there are also some studies that have investigated the effects of sea buckthorn leaves in the diet of chickens. The results of these studies include improvement in broiler performance [16,17], biochemical parameters [16], and the microbiological quality of the meat [18].

Trivalent chromium (Cr^3+^) is an essential micronutrient for all animals, which is also an important component of glucose tolerance factor. Cr could act as an indirect antioxidant, by lowering high insulin levels and preventing glucose autooxidation [19]. Diet supplementation with Cr had beneficial effects on fat metabolism [20], improved immune response [21] and alleviated heat stress [22] in broiler chickens. To our knowledge, there are no studies investigating the effect of Cr in combination with SBL on the meat quality of broilers. This study represents a novelty in the field of animal and human nutrition and food quality research by using a combination of Cr and a natural antioxidant (waste/by-products of harvest) from berry species in the diet of chickens. The results of this study address human nutrition to prevent the occurrence of metabolic diseases by improving meat with essential nutrients for humans and increasing daily nutrient intake without negative effects on food safety (oxidative lipid stability). The present study is a first research step—the development of nutrient-enriched meat—but further clinical studies need to be conducted to investigate the impacts of these products on human health.

Therefore, the aim of the present study was to evaluate whether dietary supplementation of chicken diet with Cr in combination with a natural antioxidant source (SBL) contributes positively to the nutritional quality and lipid oxidative stability of breast and thigh meat.

## 2. Materials and Methods

### 2.1. Experimental Design

A 28-day experiment (14–42 days) was conducted with 90 Cobb 500 chickens housed in an experimental shed according to the experimental protocol (no. 3511/22.05.2020) approved by the Ethics Committee of the National Research and Development Institute of Animal Biology and Nutrition, Ilfov, Romania (no. 52/30.07.2014). Chicks were fed a conventional diet until 14 days of age. Then, the chicks were divided into 3 groups, 30 chicks per group and 5 replicates with 6 birds each. Chicks were housed in three-level digestibility cages (65 × 75 × 45 cm) and raised under controlled environmental conditions. The temperature was maintained at 34 °C for the first 3 days, and then lowered by 2 °C every other week (until the thermal comfort temperature of 26 °C was reached). All cages were equipped with feeders and waterers. Experimental treatments consisted of a control treatment based on corn and soybean meal, without chromium (T0) and two additional treatments supplemented with either 0.00002% chromium (T1) or 0.00002% chromium plus 2% sea buckthorn leaves (T2). Chromium picolinate (CrPic, Santa Cruz Biotechnology, Santa Cruz, CA, USA) was used as a chromium source. Sea buckthorn leaves (*Hippophaë rhamnoides* L.) were dried, and ground as purchased from SC. SILVER ROM AGRO S.R.L., Tomești, Iasi, Romania. The supplements were added to the basal diet.

The ingredients and chemical composition of the basal diet are shown in Table 1. Feed (commercial basal diet; mash form) and water were administered on an ad libitum basis. Temperature and air relative humidity were recorded daily throughout the experimental period using a Viper Touch computer. The light regimen was 23 h light/1 h darkness. The chicks were vaccinated and after this, no medical care program or treatment was applied.

### 2.2. Broiler Performance

Throughout the experimental period (14–42 days, broiler age) the following parameters were recorded daily: body weight (g), average daily feed intake (g feed/chicken/day), average daily weight gain (g/chicken/day), and feed conversion ratio (g feed/g gain).

### 2.3. Sample Collection

Dried and ground SBL samples were used to analyze the proximate composition, fatty acid profile, bioactive nutrients (total phenolic content, total flavonoids, lutein and zeaxanthin, vitamin E and minerals), and total antioxidant capacity (TAC).

At 42 days of age, 6 chicks/group were randomly selected, electrically stunned, and slaughtered by cervical dislocation. Carcasses were eviscerated and both breast and thigh muscles and organs (liver, heart, gizzard, spleen and bile) were removed and weighed. The carcass and cuts yields were calculated based on hot carcass weight. Left breast and thigh meat samples were immediately packed into zip-top bags and stored at −80 °C for further analysis (proximate composition: dry matter, crude protein, ether extractives, ash, fatty acid profile, bioactive nutrients: total phenolic content, lutein and zeaxanthin, vitamin E, minerals), and total antioxidant capacity (TAC). The right breast and thigh meat samples were stored at 4 °C for 7 days for TBARS determination.

### 2.4. Analysis of Plant and Meat Nutrients

The analysis of proximate composition (dry matter, crude protein, ether extract, and ash) of plant and meat samples (breast and thigh) was determined according to [23]. Dry matter (ISO 6496/2001) and ash (ISO 2171/2010) were determined by the gravimetric method, crude protein (ISO 5983-2/2009) was analyzed by the Kjeldahl method, and ether extractives were performed by extraction in organic solvents (SR ISO 6492/2001).

From the extracted fat of SBL and meat samples, 0.5 g of fat was weighed, and 50 mL of acidified methanol (H_2_SO_4_ in methanol) was added and boiled under reflux for 25–30 min from the beginning of boiling. After cooling, it was transferred to a separation funnel, then 25–30 mL of distilled water and 20 mL of hexane were added. It was stirred well. The aqueous layer was then transferred to another funnel and 20 mL of hexane was added. The organic layer (hexane) was stirred and transferred to the first separation funnel. The combined hexane layers were washed twice with 10 mL of distilled water. The organic layer was dried over anhydrous Na_2_SO_4_ in a rotary evaporator. It was concentrated and the residue was again taken up in 3–5 mL of hexane and then placed in a 5 mL volumetric flask. For analysis, the extract was placed in a 1.8 mL vial. The fatty acid profile of SBL plant and breast and thigh meat samples was determined using a gas chromatography technique with a PerkinElmer Clarus 500 gas chromatograph (PerkinElmer, Inc., Waltham, MA, USA) system coupled to a flame ionization detector (FID) and capillary separation column with a high polar stationary phase TRACE TR-Fame, (Thermo Electron, Waltham, MA, USA), according to [24]. For the SBL plant, each fatty acid was expressed as g/100 g fatty acid methyl esters (FAME) and for the meat samples, each fatty acid was expressed as g/100 g fatty acid methyl esters (FAME) and as mg/100 g meat.

The fatty acid profile obtained from the chemical analysis (as g/100 g fatty acid methyl esters) was used to determine several nutritional quality indices of meat lipids. Nutritional quality indices of meat lipids were assessed by calculating the PUFA n-6/PUFA n-3, Σ PUFA/Σ MUFA ratio, atherogenicity index (IA), thrombogenicity index (IT), saturation index (SI), the ratio of hypocholesterolemic and hypercholesteremic fatty acids (h/H), and the health-promoting index (HPI) as previously described [25].

The total phenol content (TPC) was measured spectrophotometrically according to the Folin–Ciocalteu’s method from the extracts obtained previously in methanol 80% p.a., (1:10, *w*/*v*) for SBL samples and in a solution of phosphate-buffered saline (PBS) at pH 7.4 (1:10, *w*/*v*) for meat samples according to the method described previously [25]. Absorbances were recorded at 732 nm against a blank solution and the concentrations (as mg gallic acid equivalent (GAE)/g) were calculated using gallic acid as the standard solution.

Total flavonoid content was determined by the aluminium chloride colorimetric method described [26] with slight modifications. An exact volume of 1 mL of methanolic SBL extract, and 4 mL of aluminium chloride (AlCl_3_) was placed in a 10 mL volumetric flask. The solution was mixed well and left for 15 min to incubate at room temperature. After this, the absorbance of the orange-yellowish solution was measured against the blank at 410 nm using a UV-VIS spectrophotometer (JASCO V0.). The calibration curve was plotted using quercetin as the standard (R^2^ = 0.9972). The flavonoid content was expressed as mg Quercetin equivalents (QE)/per g.

Fat-soluble compounds (lutein and zeaxanthin and vitamin E) from SBL and meat samples were extracted using the method previously described by [27]. The preparation technique before the extraction included a saponification step using ethanolic potassium hydroxide solution. For this, two grams of meat samples was weighed and mixed with 130 mL of ethanol, 100 mg of butylhydroxytoluene, 2 mL of sodium ascorbate solution, 50 mg of EDTA, and 25 mL of 50% potassium hydroxide solution. Due to high concentrations of fat-soluble antioxidants in plants, only 0.5 g of dried samples were considered for the extraction described previously. Fat-soluble compounds from SBL and meat samples were analyzed using the liquid chromatographic technique (RT-HPLC) [27] with a high-performance liquid chromatograph (HPLC Finnigan Surveyor Plus, Thermo-Electron Corporation, Waltham, MA, USA). The results were expressed as µg/g.

The mineral concentrations (Cu, Fe, Mn, and Zn) were determined by flame atomic absorption spectrometry (FAAS) using a Thermo Electron—SOLAAR M6 Dual Zeeman Comfort (Cambridge, UK) according to [28]. The results were expressed as mg/kg (SBL) and µg/kg (meat samples). For a better visualization of the effects of feeding treatments over the bioaccumulation of bioactive nutrients in chicken meat, for each nutrient, the accumulation factors (AF) were calculated according to [29]. This factor is the ratio between the concentration of bioactive nutrients in meat from experimental treatments (T1, and T2) to the concentration of bioactive nutrients in meat from the control treatment (T0). The accumulation factor (AF) for each bioactive nutrient in meat (breast and thigh meat) was calculated with the following formula:

AF meat 〗_((TPC, Lutein+zeaxanthin, vit. E, Cu, Fe, Mn, Zn) )=(conc. of bioactive nutrient from experimental treatments )⁄(conc. of bioactive nutrient from the control treatment ).

### 2.5. Analysis of Antioxidant Capacity and Lipid Oxidation Status

The total antioxidant capacity (TAC) was assessed by evaluating the capacity of inhibiting the DPPH radical [30]. The calibration curve was performed using 6-hydroxy-2,5,7,8-tetramethylchroman-2-carboxylic acid (Trolox), and by plotting % DPPH inhibition depending on the concentration. The results were expressed as mmol Trolox equivalents/kg sample, describing the capacity of the samples to scavenge radicals in comparison to Trolox.

The lipid oxidative status of chicken meat was evaluated using the TBARS (thiobarbituric acid reactive substances) method [31]. The calibration curve was performed using 1,1,3,3-tetramethoxypropane hydrolyzed to an MDA stock solution. The results were expressed as µg MDA per kilogram sample (µg MDA/kg).

### 2.6. Statistical Analysis

The effects of dietary treatments on the parameters investigated in the present study were analyzed with one-way analysis of variance (ANOVA), using Addinsoft statistical software (version 2022.3.1). Graphs were drawn using Prism-GraphPad software v. 9.03 (San Diego, CA, USA). The dietary treatment groups (T0, T1, and T2) were included as fixed factors in the statistical model. Tukey’s honest significant difference test was used to separate means if there was a significant difference (*p* < 0.05). A heat map illustrating the correlation between meat quality characteristics was generated by analyzing the Pearson’s correlation coefficient test. Each cell contains the corresponding correlation and is color-coded by the correlation according to the color legend on the left, where red indicates a strong positive correlation and violet indicates a strong negative correlation. The stronger the correlation, the darker is the color.

## 3. Results

### 3.1. Chemical Composition of Sea Buckthorn Leaves (SBL)

Table 2 presents the chemical composition of dietary sea buckhorn leaves used in the diet formulation in a percentage of 2%. Regarding the proximate composition, sea buckthorn leaves have important concentrations of crude protein and crude fiber.

The results of fatty acid analysis show that sea buckthorn leaves have a high content of α-linolenic acid, with the amount of PUFA being higher than that of SFA. It is also found that the content of omega-3 fatty acids (Σ n-3) in sea buckthorn leaves is two times higher than that of omega-6 fatty acids (Σ n-6). The antioxidant profile of sea buckthorn leaves shows high content of lipophilic (vitamin E, lutein, and zeaxanthin) and hydrophilic (polyphenols) antioxidant compounds. The mineral profile shows high concentrations of Fe, Mn and Zn, and low concentrations of Cu.

### 3.2. Broiler Performance and Carcass and Cuts Yield

Table 3 shows the broiler performance and carcass and cuts yield.

No significant differences were found for broiler performance. In addition, the inclusion of Cr and Cr + SBL in the broiler diet did not significantly affect the carcass and cuts yield.

### 3.3. Proximate Composition of Chicken Meat

Table 4 shows the proximate composition of chicken breast and thigh. No significant differences were found for chicken breast. For chicken thigh, the addition of 0.00002% Cr and 2% SBL in the diet resulted in a significant increase in crude fat content compared to the other diets. In contrast, the addition of chromium alone did not significantly affect the EE content in thigh meat.

It is not only the fat content that is important, but also the quality of the fatty acids in its components, or the fatty acid profile, which is shown in the following tables (Table 5 and Table 6).

### 3.4. Fatty Acid Profile of Chicken Meat

The effects of the experimental treatments on the fatty acid composition in broiler breast meat are shown in Table 5. Although the fatty acid profile was expressed in two different ways (as g/100 g FAME and mg/100 g meat), only the results expressed in mg/100 g meat will be discussed, because this information is more relevant to the consumer. The use of Cr alone or in combination with SBL affected some of the fatty acids of the breast meat. For example, in the SFAs family, statistically significant differences were found for C15:0 acid: T2 = T1 < T0, C18:0 acid: T1 = T0 < T2, and C24:0 acid: T0 > T1 = T2. Total SFAs and PUFAs did not differ between the groups. In the MUFAs family, statistically significant differences were found for C15:1 acid: T1 = T0 < T2; C17:1 acid: T2 > T1 = T0; C22:1n9 acid: T1 < T0 = T2; and C24:1n9 acid: T2 > T1 = T0. In the T2 group, lower MUFAs (*p* = 0.0001) were found than in the T0 and T1 group.

**Table 5:** Treatment effects on the fatty acid profile of breast meat.

Fatty Acids	Treatment			SEM	*p*-value	Treatment			SEM	*p*-Value
	T0	T1	T2			T0	T1	T2		
	g/100 g FAME					mg/100 g meat				
C14:0	0.60	0.42	0.48	0.018	0.161	8.84	6.49	6.94	1.120	0.285
C15:0	0.13	0.10	0.12	0.010	0.951	1.98 ^a^	1.60 ^b^	1.81 ^ab^	0.261	0.048
C16:0	18.08	18.06	18.07	0.067	0.168	266.72	269.58	279.35	0.300	0.194
C17:0	0.22 ^b^	0.22 ^b^	0.20 ^a^	0.014	0.001	3.06	3.19	2.93	0.078	0.113
C18:0	7.57	7.76	8.20	0.117	0.139	111.14 ^a^	115.76 ^ab^	123.01 ^b^	0.241	0.022
C24:0	0.25 ^a^	0.21 ^b^	0.22 ^b^	0.012	0.0001	3.77 ^a^	3.28 ^b^	3.35 ^b^	0.268	0.033
**Σ Saturated fatty acids**	**26.85**	**26.77**	**27.29**	**0.488**	**0.090**	**394.41**	**400.30**	**420.29**	**0.294**	**0.088**
C14:1	0.09 ^a^	0.08 ^ab^	0.07 ^b^	0.124	0.001	1.17	0.97	0.91	0.290	0.140
C15:1	0.55	0.63	0.71	0.181	0.121	8.19 ^b^	9.53 ^ab^	12.17 ^a^	0.912	0.026
C16:1	2.52 ^a^	2.47 ^ab^	2.39 ^b^	0.741	0.0001	37.21	36.95	35.89	0.902	0.551
C17:1	0.12 ^b^	0.15 ^b^	0.21 ^a^	0.524	0.0001	1.93 ^b^	2.40 ^b^	3.16 ^a^	0.197	0.003
C18:1	29.45 ^a^	29.62 ^a^	27.90 ^b^	0.185	0.0001	433.22	441.99	418.79	9.501	0.336
C22:1n9	0.08 ^a^	0.06 ^b^	0.09 ^a^	0.125	0.003	1.37 ^a^	0.97 ^b^	1.36 ^a^	0.082	0.009
C24:1n9	1.02 ^b^	1.07 ^b^	1.38 ^a^	0.580	0.0001	15.19 ^b^	16.08 ^b^	20.67 ^a^	0.137	<0.0001
**Σ Monounsaturated fatty acids**	**33.81 ^a^**	**34.01 ^a^**	**32.84 ^b^**	**0.125**	**0.0001**	**497.42 ^a^**	**508.89 ^a^**	**492.96 ^b^**	**0.368**	**0.007**
C18:2n6	32.41	32.34	32.23	0.294	0.125	476.65	482.51	468.73	0.341	0.707
C18:3n6	0.27	0.29	0.27	0.325	0.230	4.21	4.51	4.28	0.322	0.394
C20:2n6	0.25 ^a^	0.21 ^ab^	0.17 ^b^	0.101	0.0001	3.87 ^a^	3.28 ^a^	2.50 ^b^	0.175	0.001
C20:3n6	0.80	0.85	0.91	0.045	0.321	11.67 ^b^	10.44 ^c^	13.70 ^a^	0.132	<0.0001
C20:4n6	2.74 ^b^	2.88 ^b^	3.64 ^a^	0.370	0.0001	38.75 ^b^	43.20 ^b^	54.59 ^a^	1.304	<0.0001
C22:2n6	0.15	0.15	0.16	0.451	0.355	2.37	2.25	2.71	0.298	0.182
C22:3n6	0.16	0.17	0.16	0.604	0.196	1.96	2.50	1.96	0.294	0.160
C22:4n6	0.33 ^b^	0.29 ^b^	0.43 ^a^	0.725	0.0001	4.97 ^b^	4.48 ^b^	6.48 ^a^	0.117	<0.0001
**Σ n-6**	**37.25 ^b^**	**37.07 ^b^**	**37.97 ^a^**	**0.147**	**0.0001**	**547.34**	**553.17**	**554.95**	**0.381**	**0.917**
C18:3n3	0.67 ^a^	0.58 ^ab^	0.53 ^b^	0.021	0.010	10.10 ^a^	8.75 ^ab^	8.06 ^b^	0.247	0.028
C18:4n3	0.23 ^b^	0.29 ^a^	0.36 ^a^	0.001	0.0001	2.84 ^c^	4.53 ^b^	5.45 ^a^	<0.0001	<0.0001
C20:3n3	0.49 ^b^	0.47 ^b^	0.63 ^a^	0.009	0.0001	7.32 ^b^	7.24 ^b^	9.51 ^a^	0.147	0.0001
C20:5n3	0.21 ^a^	0.19 ^b^	0.18 ^b^	0.002	0.0001	3.26 ^a^	2.55 ^b^	2.65 ^b^	0.207	0.005
C22:5n3	0.10 ^b^	0.11 ^b^	0.15 ^a^	0.001	0.0001	1.66 ^b^	1.78 ^b^	2.27 ^a^	0.079	0.001
C22:6n3	0.05 ^b^	0.06 ^b^	0.10 ^a^	0.002	0.0001	1.08 ^b^	1.08 ^b^	1.52 ^a^	0.096	0.012
**Σ n-3**	**1.75 ^b^**	**1.70 ^b^**	**1.95 ^a^**	**0.029**	**0.0001**	**26.30 ^b^**	**25.93 ^b^**	**29.46 ^a^**	**0.227**	**0.009**
**Σ Polyunsaturated fatty acids**	**39.05**	**38.77**	**39.92**	**0.153**	**0.158**	**573.64**	**579.11**	**584.40**	**0.379**	**0.865**
Other fatty acids	0.23	0.23	0.22	0.074	0.111	3.33	3.42	3.26	0.351	0.950

T0: control diet; T1: treatment with 0.00002% Cr; T2: experimental diet supplemented with 0.00002% Cr +2% sea buckthorn leaves (SBL); ^a,b,c^ Means within a row with no common superscript differ (*p* < 0.05); and SEM = standard error of the mean.

The following relationships were noted in the n-6 fatty acid family: C20:2n6 acid: T2 T1 = T0, C20:3n6 acid: T2 > T1 < T0, C20:4n6 acid: T2 > T1 = T0; and C22:4n6 acid: T2 > T1 = T0.

A higher content (*p* = 0.0001) of n-3 PUFAs was measured in the breast meat of chickens from the T2 group compared to the other two groups. In the n-3 fatty-acids family, statistically significant differences were found for α-linolenic acid (C18:3n3): T1 = T2 < T0; C18:4n3 acid: T2 < T1 = T0; C20:3n3 acid: T2 > T1 = T0; C20:5n3 acid: T0 > T1 = T2; C22:5n3 acid: T2 > T1 = T0; and C22:6n3 acid: T2 > T1= T0.

**Table 6:** Treatment effects on the fatty acid profile of thigh meat.

Fatty Acids	Treatment			SEM	*p*-Value	Treatment			SEM	*p*-Value
	T0	T1	T2			T0	T1	T2		
	g/100 g FAME					mg/100 g meat				
C14:0	0.49	0.47	0.49	0.032	0.188	18.36 ^b^	17.15 ^b^	22.16 ^a^	0.217	0.008
C15:0	0.18	0.18	0.19	0.011	0.063	7.15	5.64	6.69	0.272	0.249
C16:0	19.13 ^ab^	18.87 ^b^	19.99 ^a^	0.720	0.040	703.12 ^ab^	699.40 ^b^	888.05 ^a^	0.128	<0.0001
C17:0	0.22 ^b^	0.24 ^ab^	0.26 ^a^	0.002	0.0001	8.22 ^b^	8.73 ^ab^	11.36 ^a^	0.120	<0.0001
C18:0	7.15 ^b^	7.96 ^a^	7.31 ^b^	0.036	0.0001	262.78 ^c^	295.24 ^b^	325.01 ^a^	0.145	<0.0001
**Σ Saturated fatty acids**	**27.44**	**27.69**	**28.52**	**0.933**	**0.061**	**1008.93 ^b^**	**1026.15 ^b^**	**1267.62 ^a^**	**0.109**	**<0.0001**
C14:1	0.09	0.07	0.07	0.005	0.324	3.56	3.06	3.28	0.290	0.648
C15:1	0.92	0.84	0.89	0.015	0.231	33.34	30.78	39.37	0.233	0.025
C16:1	3.32 ^a^	2.94 ^b^	3.15 ^ab^	0.028	0.0001	122.46 ^b^	109.45 ^c^	140.12 ^a^	0.147	<0.0001
C17:1	0.17 ^b^	0.14 ^b^	0.24 ^a^	0.025	0.002	5.79 ^b^	4.79 ^b^	10.55 ^a^	0.170	0.0001
C18:1	29.40 ^a^	29.41 ^a^	28.31 ^b^	0.215	0.0001	1088.74 ^b^	1089.95 ^b^	1259.48 ^a^	0.157	<0.0001
C22:1n9	0.08	0.07	0.07	0.002	0.125	2.63	2.41	2.97	0.280	0.392
C24:1n9	0.58 ^b^	0.87 ^a^	0.74 ^ab^	0.022	0.025	21.58 ^b^	32.61 ^a^	33.19 ^a^	0.221	0.011
**Σ Monounsaturated fatty acids**	**34.56 ^a^**	**34.34 ^a^**	**33.48 ^b^**	**0.124**	**0.0001**	**1278.11 ^b^**	**1273.05 ^b^**	**1488.96 ^a^**	**0.139**	**<0.0001**
C18:2n6	31.26	31.16	30.92	0.122	0.185	1150.38 ^b^	1154.51 ^b^	1375.25 a	0.128	<0.0001
C18:3n6	0.20 ^b^	0.22 ^ab^	0.25 ^a^	0.004	0.0001	7.83 ^b^	8.46 ^b^	11.32 ^a^	0.141	<0.0001
C20:2n6	0.29	0.27	0.28	0.006	0.077	10.36	9.80	7.88	0.275	0.292
C20:3n6	0.49	0.50	0.45	0.003	0.233	18.07 ^b^	19.96 ^ab^	22.32 ^a^	0.232	0.023
C20:4n6	2.82	2.84	2.80	0.583	0.987	103.68 ^b^	115.12 ^ab^	122.88 ^a^	0.233	0.025
C22:2n6	0.18	0.19	0.21	0.019	0.088	6.91	6.49	9.19	0.246	0.056
C22:3n6	0.16 ^c^	0.32 ^a^	0.23 ^b^	0.143	0.0001	5.55 ^c^	19.96 ^a^	10.10 ^b^	0.079	<0.0001
C22:4n6	0.22	0.24	0.24	0.150	0.058	6.90 ^b^	7.33 ^b^	10.53 ^a^	0.212	0.006
**Σ n-6**	**35.62**	**35.74**	**35.38**	**0.350**	**0.125**	**1309.68 ^b^**	**1324.67 ^b^**	**1569.47 ^a^**	**0.136**	**<0.0001**
C18:3n3	0.57 ^a^	0.52 ^b^	0.54 ^ab^	0.002	0.0001	19.52 ^b^	18.68 ^b^	23.10 ^a^	0.415	<0.0001
C18:4n3	0.35	0.34	0.29	0.021	0.241	13.86	11.93	13.57	0.264	0.158
C20:3n3	0.34	0.35	0.40	0.084	0.711	12.59	13.58	17.85	0.252	0.082
C20:5n3	0.43	0.49	0.47	0.018	0.115	16.11	18.81	13.39	1.765	0.129
C22:5n3	0.11	0.09	0.07	0.005	0.065	4.23	4.30	3.70	0.284	0.484
C22:6n3	0.07 ^ab^	0.05 ^b^	0.10 ^a^	0.007	0.026	2.51 ^b^	2.01 ^b^	4.47 ^a^	0.213	0.006
**Σ n-3**	**1.87**	**1.84**	**1.87**	**0.025**	**0.624**	**56.73 ^b^**	**68.45 ^b^**	**78.90 ^a^**	**0.142**	**<0.0001**
**Σ Polyunsaturated fatty acids**	**37.50**	**37.58**	**37.24**	**0.551**	**0.175**	**1380.98**	**1393.12**	**1645.55**	**0.259**	**0.122**
Other fatty acids	0.50 ^a^	0.40 ^a^	0.76 ^b^	0.071	0.234	13.17 ^b^	17.79 ^b^	44.76 ^a^	0.125	<0.0001

T0: control diet; T1: treatment with 0.00002% Cr; T2: experimental diet supplemented with 0.00002% Cr +2% sea buckthorn leaves (SBL); ^a,b,c^ Means within a row with no common superscript differ (*p* < 0.05); and SEM = standard error of the mean.

Table 6 shows the fatty acid profile of thigh meat. Total PUFAs did not differ between groups. In the SFAs family, statistically significant differences were found for C14:0 acid: T2 > T1 = T0, C16:0 acid: T2 = T1= T0, C17:0 acid: T2 > T1 = T0, and C18:0 acid: T2 > T1 = T0. A higher content (*p* = 0.0001) of MUFAs, n-3 PUFAs and n-6 PUFAs was measured in the thigh meat of chickens from the T2 group compared to T1 and T0 groups. In the MUFAs family, statistically significant differences were found for C16:1 acid: T2 > T0 > T1; C17:1 acid: T2 > T0 = T1; C18:1 acid: T2 > T0 = T1; and C24:1n9 acid: T0 < T1 = T2. The following relationships were noted in the n-6 fatty acid family: C18:2n6 acid: T2 > T0 = T1; C18:3n6 acid: T2 > T0 = T1; C20:3n6 acid: T1 = T2 > T0; C20:4n6 acid: T1 = T2 > T0; C22:3n6 acid: T1 > T2 > T0; and C22:4n6 acid: T2 > T0 = T1. In the n-3 fatty-acids family, statistically significant differences were found for C18:3n3 acid: T2 > T1 = T0; and C22:6n3 acid: T2 > T1 = T0.

### 3.5. Nutritional Quality Indices of Meat Lipids

**Figure 1:**
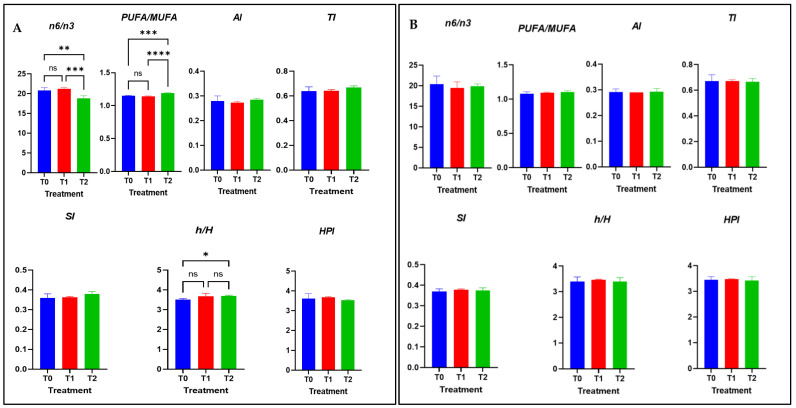
Treatment effects on nutritional quality indices of the lipids in breast (**A**) and thigh meat (**B**) The main effects of diet are presented in each graph (Prism Graph 9.03). Data are presented as mean SEM (n = 6 broilers/group). Asterisks denote statistical significance (*p* > 0.1234 ns, * *p* ≤ 0.0332, ** *p* ≤ 0.0021, *** *p* ≤ 0.0002, **** *p* < 0.0001); T0: control diet; T1: treatment with 0.00002% Cr; T2: experimental diet supplemented with 0.00002% Cr +2% sea buckthorn leaves (SBL); AI = atherogenic index; TI = Thrombogenicity index; SI= saturation index; h/H = Hypo/hypercholesterolemic index; and HPI = health-promoting index. ns= non-significant.

Figure 1 shows the effects of treatment on the nutritional quality indices of lipids in breast and thigh meat. With respect to breast meat (Figure 1A), lower n-6/n-3 and PUFA/MUFA ratios were observed in the T2 group compared with T0 and T1.

However, the h/H ratio was significantly higher in the breasts of the T2 group than in those of the T0 group. AI, TI, SI, and HPI values did not show significant differences between treatments. For thigh meat, nutritional quality indices (Figure 1B), PUFA n-6/n-3, PUFA/MUFA ratio, AI, TI, h/H, SI, and HPI values did not show significant differences between the groups.

### 3.6. Bioactive Nutrient Content of Breast and Thigh Meat

**Figure 2:**
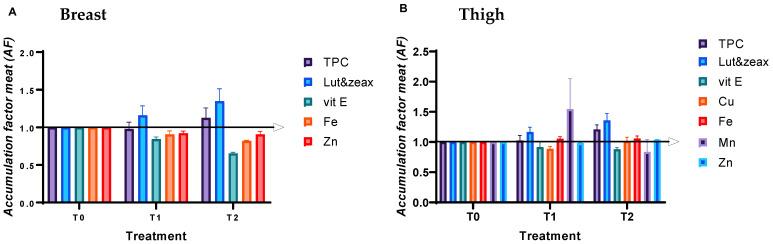
Bioactive nutrients accumulation factors (AF) in chicken breast (**A**) and thigh (**B**) under the influence of different treatments (error bar represents a range of 95% confidence). T0: control diet; T1: treatment with 0.00002% Cr; and T2: experimental diet supplemented with 0.00002% Cr +2% sea buckthorn leaves (SBL).

Figure 2 shows the accumulation factors (AF) of bioactive nutrients in chicken breast (A) and thigh (B) under the influence of different treatments. The increased intake of xanthophylls due to the addition of sea buckthorn leaves in the diet resulted in an increase in the concentrations of lutein and zeaxanthin in the chicken breast and thigh of group T2 compared to group T0 (*p* = 0.012).

The accumulation of vitamin E was lower in the breast and thigh meat of chickens fed T1 and T2 treatments compared to T0 (*p* < 0.000). Moreover, the TPC accumulation factor increased in the thigh meat of T2 compared to T1 and T0 (*p* = 0.0001), without the same trend in the breast meat (*p* = 0.06).

Regarding the mineral profile, the accumulation factor of Fe and Zn was significantly lower in the breasts of group T2 compared to other treatments (*p* = 0.0001; *p* = 0.001). However, in the thigh meat, the content of Fe and Zn was higher in T2 than in T0. Zinc content decreased in T1 compared to T0 (*p* = 0.0001). Copper and manganese content were below the method detection limit in the breast samples; in thigh meat, copper content showed no significant difference between treatments, but the manganese level was higher in T1 than in the other treatments (*p* = 0.004).

### 3.7. Antioxidant and Lipid Oxidative Stability of Chicken Meat

**Figure 3:**
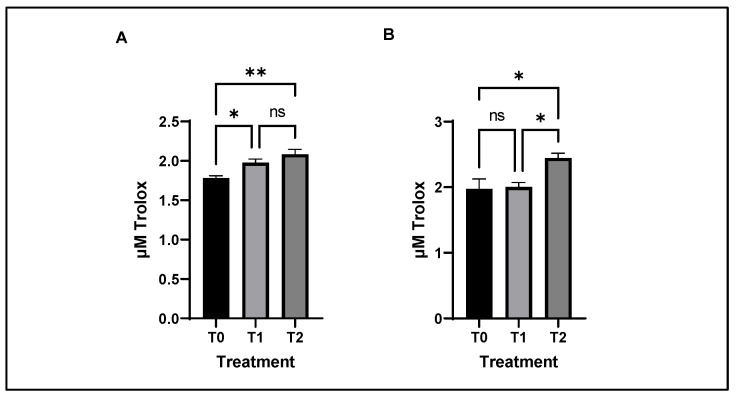
Bar graph illustrating total antioxidant capacity (TAC) as µg Trolox of chicken breast (**A**) and thigh (**B**). Asterisks denote statistical significance (*p* > 0.1234 ns, * *p* ≤ 0.0332, ** *p* ≤ 0.0021); T0: control diet; T1: treatment with 0.00002% Cr; T2: experimental diet supplemented with 0.00002% Cr +2% sea buckthorn leaves (SBL); ns = non-significant.

The effect of feeding Cr alone or in combination with SBL on TAC of chicken breast and thigh meat is shown in Figure 3.

The breast samples from T2 and T1 showed a higher antioxidant capacity compared to those from T0 (*p* = 0.005). However, in the thigh meat, TAC showed a higher activity only in group T2 than in T1 and T0.

**Figure 4:**
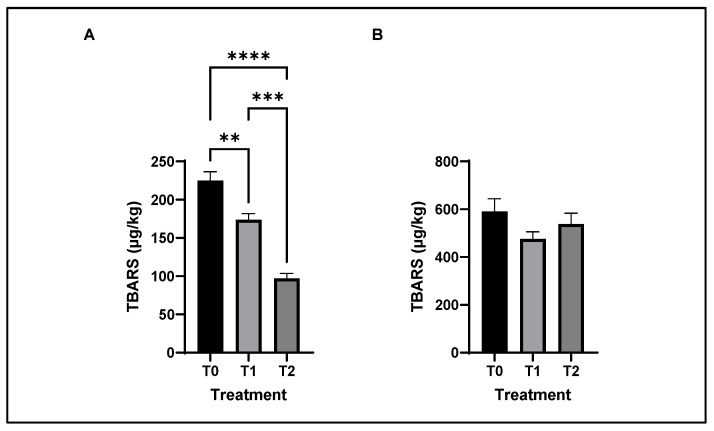
Bar graph illustrating lipid oxidation status (as TBARS, µg MDA/kg) of chicken breast (**A**) and thigh (**B**) after 7 days of storage. Asterisks denote statistical significance (** *p* ≤ 0.0021, *** *p* ≤ 0.0002, **** *p* < 0.0001); T0: control diet; T1: treatment with 0.00002% Cr; T2: experimental diet supplemented with 0.00002% Cr +2% sea buckthorn leaves (SBL), ns = non-significant.

The effect of feeding Cr alone or in combination with SBL on the TBARS of chicken breast and thigh meat is shown in Figure 4. Feeding Cr alone or in combination with SBL in chicken diets decreased the TBARS concentration in breast meat after 7 days of refrigeration storage. However, the treatments had no effect on TBARS in thigh meat.

### 3.8. The Relationship between Meat Characteristics Given by Pearson Correlation Matrix

**Figure 5:**
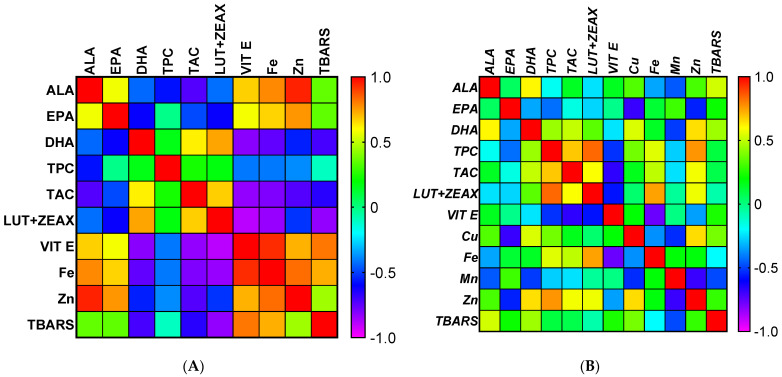
Heatmaps representing the correlation between meat quality characteristics in breast (**A**) and thigh meat (**B**). Each cell contains the corresponding correlation and is color-coded by the correlation according to the color legend on the left, where red indicates a strong positive correlation and violet indicates a strong negative correlation. The stronger the correlation, the darker is the color. Abbreviations: ALA—alfa-linolenic acid; EPA—eicosapentaenoic acid; DHA—docosahexaenoic acid; TPC—total phenolic content; TAC—total antioxidant capacity; and TBARS—thiobarbituric reactive species. T0: control diet; T1: treatment with 0.00002% Cr; and T2: experimental diet supplemented with 0.00002% Cr +2% sea buckthorn leaves (SBL).

Figure 5 shows the correlations between the different meat quality items. To account for multiple testing, only those differences that have *p*-values < 0.05 are discussed.

In breast meat (Figure 5A), ALA was positively correlated with EPA (*p* < 0.05), vitamin E, Zn, and negatively correlated with TPC and TAC. In addition, TAC was negatively correlated with Fe, Zn, and TBARS and positively correlated with DHA. As shown in Figure 4, EPA was negatively correlated with DHA, lutein, and zeaxanthin and positively correlated with vitamin E, Fe, and Zn. As for the content of DHA, a positive correlation was found with TAC, lutein, and zeaxanthin and a negative correlation with vitamin E, Fe, and TBARS. Lutein and zeaxanthin were negatively correlated with EPA, vitamin E, Fe, and TBARS and positively correlated with DHA and TAC. Regression analysis showed a positive correlation of zinc with vitamin E, Fe, and EPA and a negative correlation with TAC. TBARS was found to be negatively correlated with DHA, TAC, lutein, and zeaxanthin and positively correlated with vitamin E and Fe.

In thigh meat (Figure 5B), ALA was positively correlated with TBARS and DHA. In addition, TBARS was negatively correlated with Mn. A negative correlation was found between DHA and Mn and a positive correlation between DHA and TAC, Cu, and Zn. EPA was negatively correlated with Cu (*p* = 0.0016) and Zn (*p* = 0.018). Regression analysis showed a negative correlation between TPC and vitamin E and a positive correlation between TPC, TAC, lutein and zeaxanthin, Fe, and Zn. In addition, TAC was positively correlated with DHA, TPC, lutein, and zeaxanthin, and Zn. Lutein and zeaxanthin were negatively correlated with vitamin E, whereas they were positively correlated with TPC, TAC, Fe, and Zn. A negative correlation was found between vitamin E and TPC, lutein, and zeaxanthin and Fe. Cu was negatively correlated with Mn and EPA and positively correlated with DHA and Zn. Regression analysis showed a negative correlation between Fe and vitamin E and a positive correlation with TAC, TPC, lutein, and zeaxanthin. Mn was negatively correlated with DHA, Cu, Zn, and TBARS. With respect to Zn, significant negative correlations were observed with EPA and Mn and a positive correlation with DHA, TPC, TAC, lutein and zeaxanthin, and Cu.

## 4. Discussion

### 4.1. Chemical Composition of Sea Buckthorn Leaves (SBL)

Analysis of the nutrient profiles of sea buckthorn leaves (SBL) has shown that they are rich in bioactive antioxidant compounds, such as hydrophilic antioxidants (polyphenols) and lipophilic antioxidants (xanthophyll, vitamin E) and microelements, especially Fe. Due to their high content of phenols and carotenoids, the studied sea buckthorn leaves have a high antioxidant capacity (demonstrated by the ability to scavenge DPPH free radical). Many other studies showed the greatest nutritional value of sea buckthorn leaves and reported high concentrations of polyphenols ranging from 41.60 to 103 mg GAE/g [32,33], flavonoids content ranging from 5.63 to 14.37 mg rutin equivalent/g [34], vitamin E, 54.1–659.0 mg/kg [35,36], carotenoids, 3.5–4.2 mg/100 [37], and antioxidant capacity ranging from 123.47 to 138.72 mg Trolox equivalent/g [33]. All previous results from the literature are consistent with those in this study.

Recent studies have shown that sea buckthorn leaf extract has a high concentration of polyphenols and a high antioxidant capacity (expressed as mg Trolox equivalent/g) [11,33]. Other researchers [9] have shown that the lipophilic antioxidants presented in SBL account for 3–8% of the total antioxidant activity of the extract. The wide range between the results of studies in the literature is due to many factors, including genetic characteristics, climate, soil conditions, plant maturity, harvest time, drying method, time, and temperature [38]. The data from the present study showed that SBL are also a rich source of minerals, especially Fe, Mn, and Zn. The results are consistent with those of other studies [39,40]. In addition, there is evidence that sea buckthorn leaves contain a similar concentration of minerals as the fruits [33].

In addition to their valuable antioxidant profile, sea buckthorn leaves have a considerable content of α-linolenic acid (ALA), a higher concentration of PUFA than of SFA, and a double concentration of Σ n-3 fatty acids than of Σ n-6. Other authors [35] showed a significant concentration of ALA, but higher (51.1 %) than in our study.

### 4.2. Broiler Performance and Carcass and Cuts Yiled

Supplementation with Cr or Cr and SBL to broilers diets did not influence the body weight, average daily feed intake, average body weight gain, or feed conversion ratio of broiler. The carcass quality and organ development were not affected by dietary supplementation with Cr and SBL. Organic chromium in the form of chromium picolinate (CrPic) was often used as a feed additive in previous studies involving the effect of chromium on the broiler growth performance. Some researchers found broilers fed supplemental CrPic showed a positive effect on growth performance [41] and others, whose results are in agreement with those of our study, showed no effect [42]. To our knowledge there have been no studies investigating the effects of the use of both Cr and SBL supplements in broiler diet on broiler performance and carcass and organ development.

### 4.3. Proximate Composition of Chicken Meat

The proximate composition of breast meat was not affected by dietary supplementation with Cr (T1) or Cr and SBL (T2). These results are in agreement with those reported by [43] which showed no effect on meat crude fat when Cr was supplemented in broiler diets.

In chicken thigh meat, the addition of Cr and SBL in the diet results in a significant increase in the crude fat content compared to the other treatments. Moreover, in addition to the amount of fat, the quality is also important. Data on the effects of sea buckthorn leaves on the proximate composition of broiler meat are lacking. However, crude fat content in *Longissimus thoracis* was increased in lambs fed sea buckthorn pomace [44]. A literature study in mice showed that SBL, especially its flavonoid glycosides, prevented adiposity and dyslipidemia by suppressing lipogenesis and dietary fat absorption [45]. Although no significant differences were observed in this study, others [46] observed an improvement in the protein content of breast and thigh meat of broiler chickens fed sea buckthorn leaves, pulp, and oil (1000 ppm sea buckthorn leaf extract, 400 ppm sea buckthorn pulp, and 0.5 mL/kg sea buckthorn seed oil). Data from the literature have shown that the sole administration of Cr in chicken feed reduces the crude fat composition of the meat [47,48,49].

### 4.4. Fatty Acid Profile of Chicken Meat

Fatty acid composition is of great importance for the nutritional quality, flavor, and oxidative stability of chicken meat, since the degree of unsaturation in the fat is critical for the susceptibility to oxidation. In the present study, the fatty acid composition could be effectively changed by the addition of Cr and SBL. The addition of Cr and SBL to the diet increased the total saturated fatty acid (Σ SFA) content, especially C14:0, C16:0, C17:0, and C18:0 in thigh muscles, while no difference was observed in breast meat. As can be seen from the above results, the association of Cr with SBL stimulated lipogenesis. The concentration of monounsaturated fatty acids (Σ MUFA) and total omega 3 polyunsaturated fatty acids (Σ n-3) expressed in mg/100 g of meat were significantly higher in the breast and thigh meat in response to Cr and SBL treatment. In fact, the Cr + SBL diet effectively increased the concentration of Σ n-6 PUFA in thigh meat, whereas no difference was observed in breast meat. The results from the present study indicate that the fatty acid profile in thigh meat is partially different from that in breast meat. This observation suggests that nutritional regulation may be different in the breast and thigh muscles. However, some authors [50] have shown that the difference could also be due to the structure and functions of the breast and thigh tissues. In our study, Σ n-6 PUFA were preferentially deposited in the thigh rather than in the breast meat, regardless of the dietary treatment. These results could be due to the different lipid composition of these two tissue types; the breast is composed of phospholipids, whereas the thigh contains triglycerides. According to several authors [50,51], n-3 PUFA are preferentially accumulated in phospholipids, but in our study n-3 PUFA were found in greater amounts in the thigh muscle (which is a storage tissue composed mainly of triglycerides) regardless of the treatment applied, than in the breast muscle (a storage tissue composed mainly of phospholipids). Similar results were first reported by [50] for adipose tissue which, despite being a storage tissue composed mainly of triglycerides, preferentially accumulated n-3 PUFA. The exact distribution of fatty acids is rather unclear and not generally valid. According to some researchers [50,52], situations are conceivable in which fatty acids may be redistributed to other tissue types than expected in response to metabolic demand.

Although α-linolenic acid (ALA) had significantly lower concentrations in the breasts of group T2 compared with that of group T0, the long-chain polyunsaturated fatty acid (LC-PUFA), and docosahexaenoic acid (C22:6n-3, DHA), increased significantly in these groups. The concentration of ALA was significantly higher in the thigh meat of T2 compared with T0, but similarly to breast meat, the concentration of DHA increased. In this context, some authors [53] have shown that diet manipulation is an effective tool to improve the conversion of ALA to n-3 LC-PUFA by exploiting the metabolism of birds. LC-PUFA is formed from ALA through a series of reactions, such as chain elongation, and desaturation of ALA. According to some authors [54], dietary fatty acids are competitively metabolized, showing a preference for n-3 PUFA over n-6 PUFA. As indicated by our results, diets containing Cr and SBL increased the concentrations of C22:5 n-3 (DPA), C22:6 n-3 (DHA), and n-3 PUFA in the thigh muscles of chickens compared with the control treatment (T0). This trend of improving DHA content in meat is important because it is essential for brain tissue growth and function, especially during development and infancy [55], reduces the incidence of cardiovascular disease and preterm birth, protects against allergies, boosts immunity, and improves cognitive function [56]. A poultry product enriched with DHA that redresses nutritional imbalances in today’s diet and meets associated nutritional health requirements is attractive to the modern, educated consumer [57]. The current research is already taking steps to deepen the topic of meat enrichment with LC-PUFA, especially DHA, because according to current data, the bioconversion rate of ALA (abundant in vegetable oils) to DHA in the human body is extremely low [56], with most of ALA β-oxidized, and therefore DHA-rich foods and DHA supplements are the two main exogenous sources used to obtain additional DHA needed for the biological functions of the human body. In addition, the current study found that Cr and SBL supplemented diets effectively converted ALA to DHA, which cannot be synthesized in the body but is required for maintaining optimal human health and nutrition, and its storage in selected tissues (breast and thigh). The main reason for this could be that Cr, together with SBL, has antioxidant properties that could contribute significantly to the protection against peroxidation of oxidative-labile PUFA. In the context of a healthy diet and to reduce the risk of heart disease or death, the recommendation for n-3 LC-PUFA in the diet is about 250 mg per day [5,58]. For example, with one serving (150 g each) of breast meat from groups T1 and T2 in a standard meal, we can achieve n-3 PUFA intakes levels of 38.89 mg and 44.19 mg, respectively. This means that by eating a 150 g serving of chicken breast, up to 17.67% and 15.55% of PUFA n-3 of the recommended daily intake of n-3 PUFA is covered. These amounts are small compared to LC-PUFA sources such as seafood, but it is certainly interesting, considering that these meats are not a source of fat, as they have a low fat content.

### 4.5. Nutritional Quality Indices of Meat Lipids

Usually, the nutritional value of meat and the health suitability of meat fat for human consumption are evaluated by some nutritional indices (PUFA/Σ MUFA, Σ n-6/ Σ n-3, AI, TI, SI, h/H). In the current study, the higher PUFA/MUFA ratio and lower n-6/n3 ratio in the breast meat of chickens receiving SBL and Cr indicated their health-promoting effects. Meat with a lower n-6/n-3 ratio is more desirable for reducing the risk of many chronic diseases [59].

In this study, dietary supplementation with SBL and Cr increased the h/H ratio in breast meat, confirming the theory that dietary antioxidants are effective in lowering the level of hypercholesterolemic fatty acids, resulting in an improved fatty acid pattern in storage tissues [60]. In addition, the prevention of adiposity and dyslipidemia was found to be due to the high flavonoid glycoside content, including isorhamnetin 3-glucoside and quercetin 3-glucoside of SBL [61,62]. Other theories pointed out by researchers showed that various parts of sea buckthorn (the seeds, pulp, peel, and leaves) contain phytosterols that have anticholesterolemic effects in chickens by reducing hepatic cholesterol formation through decreasing the activity of enzymes, such as 3-hydroxy-3methyle glutaryl co enzyme A reductase [63,64]. In addition, there was convincing evidence that supplementation with sea buckthorn seeds (2 and 3 g/kg) improved egg quality and cholesterol levels in Rhode Island Red × Fayoumi laying hens [65].

Both AI and TI characterize the atherogenic potential of fatty acids; the lower the values of AI and TI, the greater the proportion of anti-atherogenic fatty acids present in meat. Myristic and palmitic acids are among the most atherogenic agents, whereas stearic acid is considered neutral in terms of atherogenicity but thrombogenic [66,67]. In our study, no differences were observed between treatments for myristic and palmitic acid, which was reflected in the results of AI, and TI. Moreover, the indices h/H, SI and HPI were not affected by dietary supplementation.

### 4.6. Bioactive Nutrient Content of Breast and Thigh Meat

The shift in consumer dietary and lifestyle habits toward healthy eating is now a reality. The quality of chicken meat can be easily improved for nutrients with certain functional properties by dietary manipulations [53]. Feeding chickens a combination of SBL and Cr improved fat-soluble antioxidant compounds (lutein and zeaxanthin) in breast and thigh meat, while water-soluble antioxidant compounds (polyphenols) increased only in thigh meat samples. This may be due to the presence of a variety of phytomolecules in SBL that are transferred to storage tissues. It is difficult to determine the combination of supplements that will lead to optimal results because the physiological effects of using plants in the diet are due to multiple chemical substances with interactions, metabolism, and complex effects. Nevertheless, vitamin E was poorly deposited in the breast and thigh meat of chickens fed T1 and T2 treatments compared to T0. Similar results were reported by [25] when Cr was supplemented in combination with creeping wood sorrel in chicken diets. However, other authors [68] showed that the vitamin E content of sea buckthorn fruit oil had no significant effect on the depostion in rat livers and attributed this effect to the high individual variability of the biological response between the animals used.

Finally, the bioaccumulation of essential metals (Fe, Cu, Mn, and Zn) in chicken meat was different, depending on the type of meat. Basically, under the influence of Cr and SBL feeding, chicken thigh meat was enriched in Zn and Fe, while in breast meat the mineral levels decreased. A similar trend was observed by [29] when feeding minerals and medicinal plant extracts. Others [69] also showed a positive effect of Cr on Fe and Zn deposition. Feeding Cr alone increased Mn level but decreased Zn content in thigh meat compared to basal treatment.

The discrepancy in mineral accumulation could be due to the antagonism between minerals or to the chemical composition of the chicken feed, which contains other chelating agents, such as amino acids and proteins from soybean and corn [29]. This fact leads to competition between polyphenols and amino acids for complexation and bioaccumulation of metals.

### 4.7. Lipid Oxidation Status of Chicken Meat

The results of the present study indicate that supplementation of Cr alone and Cr and SBL in broiler diets increases TAC levels in both breast and thigh meat samples. Both Cr and SBL are/contain antioxidant compounds involved in exogenous antioxidant defense mechanisms in living cells. Several studies reported the antioxidant capacity of SBL in vitro [33,40,70]. Although they have different antioxidant mechanisms of action, in the present study it was observed that the addition of Cr and SBL in the diet improved the antioxidant activity in tissues compared with Cr alone.

In the current study, Cr alone or together with SBL in the diet significantly and effectively reduced the lipid oxidation process in breast meat, which was confirmed by a significantly lower TBARS level after 7 days of storage. Therefore, considering that the breast meat samples from groups T1 and T2 had increased levels of DHA (particularly susceptible to oxidation), it can be assumed that SBL together with Cr protects unsaturated fatty acids from oxidative damage. This antioxidant mechanism is not unique; there are many pathways by which antioxidant compounds could exert the protective effect against the oxidative damage of PUFA: by reducing the rate of free radical chain initiation, by scavenging the initiating free radicals or by stabilizing transition metal radicals, such as copper and iron [71,72]. Chicken is the most perishable meat after fish due to its unsaturated fatty acid content [55]. The most commonly used cut of broiler chicken worldwide is breast, whose demand has continued to increase due to its high protein and low-fat content. Compared to breast (“white meat”), the thigh meat (“dark meat”) is not as highly valued by consumers because it has a higher fat content. However, since the content of vitamins, xanthophylls, and antioxidant capacity in thigh meat was increased in this study, the consumers’ concept may change, which would bring commercial benefits.

Prepared meat products or ready-to-eat products are becoming more common in both the retail and foodservice segments [73]. Technological steps in meat processing facilitate the interaction of pro-oxidants with polyunsaturated fatty acids, dilute antioxidants, and increase tissue exposure to oxygen [74]. Increasing the oxidative stability of LC-PUFA-enriched breast meat is also important from a technological, commercial, and culinary perspective. Higher oxidative stability is associated with a longer shelf life of meat. Thus, in this study, increasing the oxidative stability and antioxidant capacity of the breast, even under the conditions of LC-PUFA enrichment, has a commercial and technological advantage over thigh meat by increasing shelf life and enabling processing technology (e.g., the production of chicken patties, chicken nuggets, etc.), under conditions that may minimize the impact on oxidation.

In addition, the increase in antioxidant capacity and decrease in the oxidative stress marker TBARS in breast meat could probably be due to the different antioxidant components of SBL and Cr. There are many studies highlighting the effectiveness of SBL in retarding lipid peroxidation in vitro and in vivo models [70,75]. For example, the addition of 0.2% SBL extract to sausages after 20 days of storage resulted in significantly lower levels of TBARS than the commercial sausage mixture with nitrite and ascorbic acid [70]. Other authors [75] found that the addition of 0.3% SBL to raw ground pork improved antioxidant potential and oxidative stability for up to 9 days during refrigerated storage.

### 4.8. The Relationship between Meat Characteristics Given by Pearson Correlation Matrix

The Pearson correlation matrix shows that vitamin E, Fe, and Zn have a stronger influence on ALA and EPA deposits in breast meat than polyphenols. However, the results of the present study show that lutein and zeaxanthin contribute to an increase in DHA and TAC concentration and a decrease in the TBARS levels of breast meat. In this context, the interpretation may be that these bioactive compounds exerted a high antioxidant activity that promoted the bioconversion of ALA to DHA and protected its oxidation, as shown by the reduction of the TBARS level in breast meat. Therefore, the protective effects of the experimental diets against lipid peroxidation in stored breast meat are polyphenol-independent; similar results were observed by [76]. However, in this study, Fe induced the formation of lipid oxidation products and thus acted as a prooxidant.

In thigh meat, the TBARS was negatively correlated with Mn, indicating that Mn acts as an antioxidant. However, DHA was negatively correlated with Mn, suggesting that Mn may counteract the formation of LC-PUFA, which causes more rapid lipid oxidation. The same was true for Zn, which affected the levels of EPA and Mn in thigh meat. As in breast and thigh meat, DHA increased because of the increase in TAC, and in addition to the values observed in breast, DHA also increased in thigh meat because of the increase in Cu and Zn. The contribution of bioactive compounds to TAC was highly variable depending on the meat type (breast or thigh). The results of this study showed that lutein and zeaxanthin were the major contributors to antioxidant capacity in breast meat, whereas lutein and zeaxanthin, polyphenols, and Zn were in thigh meat. In this context, recent studies have shown that the antioxidant properties of polyphenolic compounds do not play the major role in their mode of action [76], since their bioavailability is quite low.

In thigh meat, vitamin E was negatively correlated with lutein and zeaxanthin, TPC, and Fe. Although some studies have shown that dietary polyphenols can protect vitamin E by acting as a kind of first-class antioxidant [77,78], an opposite trend was found in our study. The relationship between bioactive compounds (polyphenols, xanthophyll, etc.) and vitamin E is not easily and completely clarified and it could well be that phenols influence the redistribution of the vitamin between tissues and plasma [76]. Some authors [79] have shown that dietary antioxidants behave differently with respect to the absorption of α-tocopherol. The authors showed that naringenin can impair intestinal absorption of α-tocopherol, which can result in lower deposits in targeted tissues. It has been pointed out that bioaccessibility and uptake of fat-soluble vitamins correlate negatively with the presence of fibers, phytates, saponins or tannins [80,81]. Tannins are secondary compounds, formed in plant leaves, fruits and bark [80,82]. The negative correlation between total polyphenols and vitamin E content observed in our study could be due to the possible presence of tannins in sea buckthorn leaves. To confirm these hypotheses, further studies need to be performed to determine the concentration of tannins and to investigate the effects on vitamin E bioaccessibility.

In this study, vitamin E and lutein and zeaxanthin have antagonistic effects because they are fat-soluble compounds and probably have the same transport mechanism, so they compete for the transporters. The interactive antioxidant effects are closely related to the bioaccessibility and bioavailability of phytochemicals. This interaction between antioxidants is the result of the actual fractions of phytochemicals that are absorbed, distributed in the different cells or tissues, metabolized, and available at specific sites or targets of phytochemicals [83]. Further studies on bioavailability need to be conducted to clarify this mechanism.

## 5. Conclusions

The use of Cr in combination with a natural antioxidant source (SBL) in chicken feed resulted in meat with beneficial health properties by increasing the content of nutrients, such as xanthophylls and minerals, improving the fatty acid pattern, promoting the increase in DHA in meat, and effectively reducing their oxidation. Furthermore, lipid metabolism and antioxidant activity differed between breast and thigh meat: n-6 and n-3 PUFA deposited preferentially in thigh meat rather than in breast meat, and oxidative stability increased in breast meat.

## Figures and Tables

**Table 1:** Main ingredients and nutrient analysis of the diets (%).

Ingredient	Grower (14–28 d)			Finisher (28–42 d)		
	T0	T1	T2	T0	T1	T2
	%					
Corn	40.18	40.18	38.36	44.70	44.70	42.91
Soybean meal	26.33	26.33	26.07	21.32	21.32	21.06
Wheat	20.00	20.00	20.00	20.00	20.00	20.00
Corn gluten	5.00	5.00	5.00	5.00	5.00	5.00
Sea buckthorn leaves (SBL)	-	-	2.00	-	-	2.00
Oil	3.78	3.78	3.83	4.62	4.62	4.66
Monocalcium phosphate	1.36	1.36	1.36	1.19	1.19	1.19
Calcium carbonate	1.25	1.25	1.23	1.13	1.13	1.11
Salt	0.36	0.36	0.36	0.36	0.36	0.36
Methionine	0.30	0.30	0.31	0.26	0.26	0.27
Lysine	0.30	0.30	0.32	0.30	0.30	0.31
Threonine	0.09	0.09	0.11	0.07	0.07	0.08
Choline	0.05	0.05	0.05	0.05	0.05	0.05
Chromium	-	0.00002	0.00002	-	0.00002	0.00002
A1 Premix ^1^	1.00	1.00	1.00	1.00	1.00	1.00
Total	100	100	100	100	100	100
Chemical analysis- theoretical						
ME, Kcal/kg	3128.99			3217.72		
CP, %	21.50			20.00		
EE, %	6.01			6.49		
CF, %	3.57			3.36		
Ca., %	0.87			0.81		
P, %	0.70			0.65		
Available phosphorus, %	0.43			0.41		

^1^ 1 kg of A1 premix contains 1,100,000 IU/kg vitamin A; 200,000 IU/kg vitamin D3; 2700 IU/kg vitamin E; 300 mg/kg vitamin K; 200 mg/kg Vit. B1; 400 mg/kg vitamin B2; 1485 mg/kg pantothenic acid; 2700 mg/kg nicotinic acid; 300 mg/kg vitamin B6; 4 mg/kg Vit. B7; 100 mg/kg vitamin B9; 1.8 mg/kg vitamin B12; 2000 mg/kg vitamin C; 8000 mg/kg manganese; 8000 mg/kg iron; 500 mg/kg copper; 6000 mg/kg zinc; 37 mg/kg cobalt; 152 mg/kg iodine; and 18 mg/kg selenium. T0: control diet; T1: treatment with 0.00002% Cr; and T2: experimental diet supplemented with 0.00002% Cr +2% sea buckthorn leaves (SBL).

**Table 2:** Chemical composition of sea buckthorn leaves (SBL).

Analyzed Parameters	Sea Buckthorn Leaves (SBL)
Proximate composition (%)	
Dry matter	91.63
Crude protein	14.48
Ether extractives	5.12
Crude fiber	13.68
Ash	6.37
Fatty acids (g/100 g FAME)	
Σ Saturated fatty acids (SFA)	30.76
Σ Monounsaturated fatty acids (MUFA)	32.66
Σ Polyunsaturated fatty acids (PUFA)	35.65
Σ Unsaturated fatty acids (UFA)	68.31
SFA/UFA	0.45
PUFA/MUFA	1.09
Σ n-3 of which:	24.92
α-linolenic acid	23.61
Eicosadienoic acid	0.47
Σ n-6 of which:	10.72
Arachidonic acid	0.69
Σ n-6/ Σ n-3	0.43
Antioxidant profile	
TPC, mg/g GAE	58.61
TFC, mg/g QE	9.03
Lutein and zeaxanthin, µg/g	583.4
Vitamin E, µg/g	321.29
TAC, µM Trolox	1147.91
Mineral profile (mg/kg)	
Copper	3.05
Iron	334.79
Manganese	159.59
Zinc	126.78

FAME = fatty acid methyl esters; TPC = Total phenol content; TFC = Total flavonoid content; TAC = total antioxidant capacity; GAE = gallic acid equivalents; and QE = quercetin equivalents.

**Table 3:** Treatment effects on broiler performance and carcass and cuts yield.

Item	Treatment			SEM	*p*-Value
	T0	T1	T2		
Broiler performance					
Initial BW, g	521.65	521.47	521.65	3.59	0.9994
Final BW, g	3246.74	3244.06	3260.00	2.68	0.9542
ADWG, g/broiler/day	97.84	97.24	97.80	0.746	0.7554
ADFI, g/broiler/day	158.68	149.33	157.73	4.688	0.6747
FCR, g feed/g gain	1.61	1.54	1.62	0.044	0.7108
Carcass and cuts yield					
Carcass yield, %	84.79	81.80	81.17	0.891	0.2151
Breast yield, %	26.68	27.67	26.87	0.424	0.6446
Thigh yield, %	21.23	22.01	21.73	0.264	0.5118
Gizzard, %	1.16	1.14	1.12	0.046	0.9587
Liver, %	2.44	2.34	2.59	0.069	0.3793
Heart, %	0.57	0.54	0.54	0.018	0.8013
Spleen, %	0.13	0.12	0.13	0.007	0.8766
Bile, %	0.08	0.10	0.07	0.008	0.2576

BW: body weight; ADWG: average daily weight gain; ADFI: average daily feed intake; and FCR: feed conversion ratio.

**Table 4:** Treatment effects on the proximate composition (%) of breast and thigh meat samples.

Item	Treatment			SEM	*p*-Value
	T0	T1	T2		
Breast					
Dry matter	25.32	25.17	25.99	0.6549	0.561
Crude Protein	22.78	23.12	23.39	0.5394	0.530
Ether extractives	1.46	1.50	1.49	0.0024	0.061
Ash	1.04	1.06	1.10	0.0015	0.188
Thigh					
Dry matter	23.47	23.89	24.47	0.8651	0.346
Crude Protein	18.84	19.21	18.89	0.5478	0.762
Ether extractives	3.68 ^b^	3.70 ^b^	4.45 ^a^	0.0197	0.0001
Ash	0.85	0.88	0.91	0.0025	0.288

T0: control diet; T1: treatment with 0.00002% Cr; T2: experimental diet supplemented with 0.00002% Cr +2% sea buckthorn leaves (SBL); ^a,b^ Means within a row with no common superscript differ (*p* < 0.05); and SEM = standard error of the mean.

## Data Availability

All data are contained within the article.
